# Ethical decision making in dental education: a preliminary study

**DOI:** 10.1186/s12910-015-0046-4

**Published:** 2015-07-28

**Authors:** Mehmet İlgüy, Dilhan İlgüy, İnci Oktay

**Affiliations:** Department of Dentomaxillofacial Radiology, Yeditepe University Faculty of Dentistry, Bağdat Street, İstanbul, Turkey; Departments of Pedodontics and Public Dental Health, Yeditepe University Faculty of Dentistry, Bağdat Street, İstanbul, Turkey

**Keywords:** Ethical decision making, Dental education, HIV

## Abstract

**Background:**

In terms of ethical decision making, every clinical case, when seen as an ethical problem, may be analyzed by means of four topics: medical indications, patient preferences, quality of life, contextual features. The aim of this study was to compare the performance of 4^th^ year dental students on Ethical Decision Making before and after a course on ethics.

**Methods:**

Fourth year dental students (n = 37) from academic year 2013–2014 participated in the study. A 3-h lecture, which was about four topics approach to clinical ethical case analysis, was given to the students. The lecture was based on case scenarios related with dental ethics. After the completion of lectures,a case scenario was presented to the students to assess their ethical decision making abilities. At the end of the exam, four topics and ethical judgment were evaluated. Their performances on this examination were evaluated before and after lectures. Statistical evaluation was performed with the significance level set at *p* < 0.05.

**Results:**

A statistically significant difference was found between the means of four topics (*p* < 0.05). There was no statistically significant difference between the mean scores of judgment of ethical decision (*p* > 0.05). The mean total score of the students after the course was significantly higher than before course (67.5 and 54.4, respectively; *p* < 0.05).

**Conclusion:**

More lectures should be implemented to the curriculum to increase the student awareness of ethical issues and to reach the ultimate goals of dental education.

## Background

Medical ethics is named as a structured system, which aims to present appropriate strategies for solving the ethical problems in medical sciences and dentistry. Dentists have a responsibility to their patients and communities in which they practice [1].

Clinical cases that raise ethical problems are also a challenge to solve for the dental practitioners. When faced with difficult clinical situations, using a systematic approach can make the decision making process simple and quickly in reaching an ethical decision or recommendation [2]. American Dental Education Association reported that; dental graduates must be competent to apply ethical and legal standards in the provision of dental care [3]. To prepare dental students for solving the ethical dilemmas easily, new ethical strategies should be added to the dental curriculum. Often, the clinical facts like patient values, preferences and family factor must be also taken into account.

Previous studies reported about other approaches, which were used to guide decision-making about ethical issues [4–10]. Kaldjian et al. [10] described an approach that addresses the heterogeneity of clinical problems that at first appear ethical and acknowledges the ethical pluralism that pervades clinical ethics with six familiar sources of ethical value.

One of the ethical strategies is the four topics method, which was described by Jonsen et al. [11], was used to give clinicians with a framework for focusing on specific aspects and for connecting the circumstances of a case to their underlying ethical principles (Table 1). Each topic—medical indications, patient preferences, quality of life, and contextual features—represents a set of specific questions to be considered in working through the case [2]. Each box represents one or more of the underlying core ethical principles of medicine, which helps link the principles to the specific details of any case. The approach allows us to organize our thoughts with respect to the different aspects of a clinical case and to have a starting point to discuss conflicted areas with patients and their families. One of the reasons for selecting this method is its simplicity. Also this methodology was named as the “four quadrants” or “four boxes” approach and was popularized through its use in the ethics fellowship training program at the University of Chicago’s MacLean Center for Clinical Medical Ethics [2].

**Table 1:** Four topics approach to clinical ethics case analysis [2]

**Medical Indications**	**Patient Preferences**
• What is the patient’s medical problem? History?Diagnosis? Prognosis?	• Is the patient mentally capable and legally competent? Is there evidence of capacity?
• Is the problem acute? Chronic? Critical? Emergent? Reversible?	• If competent, what is the patient stating about preferences for treatment?
• What are the goals of treatment?	• Has the patient been informed of benefits and risks, understood this information, and given consent?
• What are the probabilities of success?	• If incapacitated, who is the appropriate surrogate? Is the surrogate using appropriate standards for decision making?
• What are the plans in case of therapeutic failure?	• Has the patient expressed prior preferences)?
• In sum, how can this patient be benefited by medical and nursing care, and how can harm be avoided?	• Is the patient unwilling or unable to cooperate with medical treatment? If so, why?
	• In sum, is the patient’s right to choose being respected to the extent possible in ethics and law?
**Quality Of Life**	**Contextual Features**
• What are the prospects, with or without treatment, for a return to normal life?	• Are there family issues that might influence treatment decisions?
• What physical, mental, and social deficits is the patient likely to experience if treatment succeeds?	• Are there provider (physician, nurse) issues that might influence treatment decisions?
• Are there biases that might prejudice the provider’s evaluation of the patient’s quality of life?	• Are there financial and economic factors?
• Is the patient’s present or future condition such that his or her continued life might be judged as undesirable?	• Are there religious or cultural factors?
• Is there any plan and rationale to forgo treatment?	• Are there limits on confidentiality?
• Are there plans for comfort and palliative care?	• Are there problems of allocation of resources?
	• How does the law affect treatment decisions?
	• Is there any conflict of interest on the part of the providers or the institution?

To our knowledge, there is no reference about evaluation of the dental students using the aforementioned four topics of ethical principles. The aim of this study was to compare the performance of 4^th^ year dental students on ethical decision making, using the four topics approach, before and after 3 h lecture on ethics.

## Methods

The study has received formal review and approval by the Yeditepe University Faculty of Dentistry Institutional Review Board. Fourth year dental students (n = 37) from academic year 2013–2014 participated in the study. All the dental students gave written informed consent to participate in the study. First, a case scenario was presented to the students, an answer sheet which contains only titles of four topics (medical indication, patient preferences, quality of life and contextual features) was given and the students were asked to solve the case using their knowledge of ethics, which they obtained from the lecture “Deontology and Ethics in Dentistry” in 3^rd^ grade, according to what they understand from these subtitles.

Then a 3-h lecture on four topics approach, used for clinical ethical case analysis, was given to the students. The lecture was based on case scenarios related with dental ethics. After the completion of the lecture, the same case scenario was presented to the students again to make an ethical decision using the four topics approach. After the presentation of the case scenario, an answer sheet, which included titles of four topics and its specific questions, was distributed to the students.

In the lecture, the “Judgment of ethical decision” was added as a 5th section. In this part, it is expected that the student can understand the problems, use objective and ethical reasoning skills, reflect on both their judgment process and their resulting decision, and planning to implement decisions [12–14].

The lecturer in charge also provided information to the students about how the responses would be scored at each level before the test. Students were allowed 50 min to complete the test. Their answers on this examination were evaluated before and after the additional ethical lectures. One marking protocol was used for each student’s response. The highest score for each category was as follows: Medical Indications: 20; Patient Preferences: 15; Quality of Life: 15; Contextual Features: 20; Judgment of Ethical Decision: 30. When added, all scores summed up to 100. An answer key was prepared for each group (Table 2). All responses were evaluated by the same lecturer.

**Table 2:** Analyze of the case according to the four topics approach and judgment of ethical decision

**Medical Indications**	**Patient Preferences**
• Define the medical status with a detailed anamnesis: HIV (human immunodeficiency virus) -positive, no activity, no major progressive diseases.	• Patient appears mentally competent and understands the implications of dental indications.
	• If the patient rejects tooth extraction, he should be informed about the possible complications.
• Dental Prognosis: a severe decay in the right mandibular second molar with the root involvement. Restorative treatment is not indicated. Tooth extraction needed.	• Family is unaware of his HIV positivity (wants not to tell to the family)
• Goal of dental treatment: Dental care should be provided according to the patient benefits. A protective dental care and recall should be applied.	• A patient consent to a dental treatment, the decision is made in a voluntary manner
**Quality of Life**	**Contextual Features**
• Patient has high level of dental pain.	• Patient is unemployed and has financial support from his mother. No problem in terms of financial issues. The patient was able to afford the extraction and prosthetic treatment afterwards.
• With appropriate treatment, he can return to normal life and has no complications about having a severe decay.	• Family is unaware of their son HIV+
	• AIDS was categorized on the list of illnesses requiring obligatory notification and seropositive means potentially infectious.
• Patient is HIV + and a serious infection may occur.	• The medical status of the patient is recorded to patient files confidentially to be shared by related faculty members.
Judgment of Ethical Decision	
• Defining and understanding the ethical problems thoroughly by analyzing the situation with general reasoning.	
• All patients have a right to privacy, especially with regard to the doctor-patient relationship. Even though AIDS was categorized on the list of illnesses requiring obligatory notification and seropositive means potentially infectious,since patient is an adult, the seropositivity should not be shared with his family without permission.	
Patient should also be informedabout possible contamination of his sexual partner.	
The student should be optimist during the management of the patient and avoid from making the patient feel discriminated.	

### The case scenario

“A 28 year old male patient presented to our clinic with a severe tooth pain. He was single and unemployed. He indicated that he was HIV (human immunodeficiency virus) -positive. He was contaminated with HIV during his appendectomy operation 3 years ago. He had no other systemic diseases. He stated that white blood cell and viral load counts were regularly being counted at the infectious disease clinic. Blood analysis results indicated that there was no activity in the HIV status. The clinical and radiological examination revealed that there was a severe decay in the right mandibular second molar. Because of the root involvement, extraction of the tooth was decided. However; the patient indicated that he wanted his tooth to be restored and wanted not to tell his family about seropositivity until they were ready to handle this information. The patient’s mother was also present at the clinic during the examination for financial support. A general consent form was received after the patient was informed about the benefits and risks of the treatment.”

SPSS 15 (Statistical Package for Social Sciences, IBM, New York, USA) for Windows® was used for statistical analysis of the data. The student-*t* test was used for the statistical evaluation and performed with the significance level set at *p* < 0.05.

## Results

Table 3 shows the mean scores of the students on ethical decision making process according to the four topics as well as judgment of ethical decision before and after the course. A statistically significant difference was found between the mean scores of medical indication, patient preferences, quality of life and contextual features (*p* < 0.05). There was no statistically significant difference between the mean scores of “judgment of ethical decision” (*p* > 0.05). The mean total score of the students after the course was significantly higher than before course (67.5 and 54.4, respectively; *p* < 0.05). Even though the scores of the students regarding the contextual features were less than the other aspects, there was significant development in students’ performance after the course (*p* < 0.05).

**Table 3:** The mean scores of the students on ethical decision-making process according to the four topics and judgment of ethical decision before and after the course. The first column indicates the highest possible score that a student can receive for each category

	Score	Score before the course Mean ± SD	Score after the course Mean ± SD	*p*
Medical Indications	20	12.3 ± 5.96	14.8 ± 4.66	*0.049*
Patient Preferences	15	12.6 ± 4.28	14.2 ± 1.87	*0.041*
Quality Of Life	15	8.27 ± 4.14	10.2 ± 2.42	*0.016*
Contextual Features	20	5.05 ± 6.18	9.7 ± 4.5	*0.038*
Judgment of Ethical Decision	30	16.2 ± 8.83	18.6 ± 6.2	*0.165*
Total Score	100	54.4 ± 18.6	67.5 ± 14.3	*0.042*

## Discussion

Dental students should have the responsibility to act in the patient’s best interest and to provide the highest standards of clinical care. Dental faculties may be already giving this instruction. Increasing awareness and the addition of content addressing diversity issues may also help support greater understanding of the role stigma can play in access to health care [15]. Although some studies suggest that it is difficult to change the attitudes and misconceptions of students towards HIV/Acquired Immune Deficiency Syndrome (AIDS) [16–18],others indicate that providing knowledge and experience has a positive effect on their attitudes [19, 20]. Patients with HIV/AIDS have challenged the ethics of the dental profession. The seriousness and infectious characteristics of the illness give rise to the occurrence of some ethical dilemmas about the treatment of such patients [11]. However, in the present study, after the lectures, the total score of ethical decision making process (67.5, Table 3) was sufficient but not very high. Rohn et al. [15], reported that there is a definite need to provide instruction and experience to dental students beyond the context of general ethical obligations. In our faculty, inviting HIV-positive individuals to speak with students to share their perspectives, as patients may be helpful to raise their understanding. But this result may be related small sample size.

The topic of medical indications crucial for helping frame the treatment options available to dentist and, in turn, the patient and family in a given case. All decisions regarding dental treatment involve a calculation regarding the potential benefits and risks of the proposed intervention(s) [2]. NaidooandVernillo [21] reportedthat it is ethically unjustified to disclose the HIV status of a patient to a referring physician whether it is necessary or not without informing the patient about such disclosure. Furthermore, the disclosure is warranted only in as much as such information will primarily lessen the risk of clinical complications for the patient and not necessarily the potential for HIV exposure to the healthcare professional. In the present study, in medical indication category, students should know that in our country, AIDS was categorized on the list of illnesses requiring obligatory notification and seropositive means potentially infectious. In our faculty, the medical status of the patient is recorded to patient files confidentially to be shared by faculty doctors. Dental care should be provided according to the patient benefits. The basic principle being the respect of the individual, a patient should not be screened without t his consent [21, 22]. In the USA, such notifications are often identified by name, but must of course remain confidential. In France, where infectious diseases are notified anonymously, debate has been raised more recently around the idea of a register, be it anonymous or not [23]. In the present case, there was no need to screen patient because patient had already the laboratory test results with himself. Also, the diagnosis was clear and the illness was not active. Thus, the students should be aware of the possible risks associated with the treatment of this specific group of patients. In the present study, this awareness was increased after the lectures related with medical indications (Table 3, *p = 0,049*).

The topic of patient preferences focuses on the expressed wishes of the patient. It is also important to evaluate the patient’s understanding of the dental indications relevant with the case. Has the patient been provided with sufficient information, and is he or she able to assimilate and use this information to formulate a statement of preferences? Equally important is to be sure that when a patient consent to a dental treatment, the decision is made in a voluntary manner [2]. In the present case, student should be aware about patient preferences about not telling his HIV status to his family. The patient was an adult and mentally capable, dentist should respect the patient’s wishes in this manner. In this particular case, there is a conflict between the patient’s treatment preferences and the dental indication. Student should know that patient must be informed about the appropriate treatment and prognosis. Our results show that students receive higher scores about patient preferences section after the lecture (Table 3, *p = 0,041*).

A major goal of medical treatment is to restore, maintain, or improve quality of life. In clinical ethics case analysis, it is important to consider what effect the indicated treatment will have on the patient’s quality of life. Respect for patient autonomy implies that the patient is best positioned to judge his own quality of life. The principles of beneficence and no maleficence help to determine the appropriateness of accepting or rejecting the potential treatment alternatives [2]. HIV-positive patients must achieve and maintain a “functional oral health status in order to receive proper nutrition, for the prevention of oral infections, and improvement of their quality of life” [24]. In the present study, students are expected to know that there is no alternative treatment due to severe decay of tooth in this case. The patient must be informed about this situation. Students should also have knowledge about “the benefit of the patient, HIV-positive or not, remains the primary goal” [25]. In this quality of life section, students were also expected to make a protective dental care. In the present study, the mean scores of this topic did not seem very high but increased after the lecture (Table 3, *p = 0,016*).

The last step in the four topics method is to think the larger perspective in which the case is occurring and to determine whether any contextual features are relevant to the case and its ethical analysis [2]. Multiple social factors, the dynamics of the family, lifestyle of the patient, and cultural and religious beliefs should be determined in this section. In the present case, there was no family effect on treatment decision of the patient because they were unaware of the patient’s special condition. Similarly, there were no financial factors, which might influence the treatment decision, as there was no problem in terms of financial issues. The patient was able to afford the extraction and prosthetic treatment afterwards. This topic was found to receive the lowest score in spite of the previous lecture given to the students (Table 3, *p = 0,038*). This implies that more emphasis should be placed on this aspect.

In the present study, the additional part to this evaluation is the Judgment of Ethical Decision; a summary and judgment of final decision should be made by the students. Fourth year students must have an ethical attitude about patients using their ethical instructions, which were given only at the 3rd year (14 h). A fourth year student must be aware of the basic ethical guidelines he needs to follow in his professional career to protect himself from situational anomalies and should be capable of collecting data, identify the ethical problem and analyze the situation.

The four topics method is one of many approaches to clinical ethical decision-making. It helps to highlight areas of controversy and to clarify the principles underlying the circumstances of a clinical ethics case. Clinical ethical decision-making is not always easy, but it can be overwhelming for clinical trainees [15]. In this study, in terms of four topics approach, there was a significant difference after the lecture. This finding suggests that such a systematical methodology was useful for students to analyze an ethical problem.

The relatively small sample size used in the present study is one of the limitations. However, the student population is relatively less in the faculty of dentistry due to the fact that it is a private institution. Although not being significant, there was an increase in the scores received from Judgment of Ethical Decision section. Since this is a preliminary study, further studies are needed to better understand whether there will be an improvement with this approach in a larger sample.

## Conclusions

Our results suggest that updating previously acquired information may be useful for the retention of knowledge. Some modifications may be done in the content of the curriculum to increase the ethical awareness of students. More lectures could be implemented to the curriculum to increase the student awareness of ethical issues and to reach the ultimate goals of dental education. Case based lectures using such systematic approaches as a means of delivery of information may be more supportive for students in order to understand the ethical problems.
